# 

**Published:** 1879-10

**Authors:** 


### CONTENTS:

ARTICLE I.—Proceedings of the Maryland and District of Columbia Dental Society.. 241
Address of Dr. T. S. Waters........................... 241
Address of Dr. J. C. Smithe........................... 245
Syphilis of the Teeth, Hair, Nails and Skin. By Martin B. Scott, M. D........................... 248
ARTICLE II.—A Few Thoughts on Filling Teeth. By T. H. Parramore, D. D. S.... 274
American Academy of Dental Science........................... 274
ARTICLE III.—Transactions of the Odontological Society of Great Britain.... .... 278

### EDITORIAL, ETC.

In the Blood........................... 284
Don't Roll your Manuscripts; and other Don'ts........................... 285

### BIBLIOGRAPHICAL.

Eyesight and How to Care for It........................... 286
Students' Pocket and Medical Lexicon........................... 286
Medical Chemistry........................... 287
A Guide to Surgical Diagnosis........................... 288
Vegetarianism the Radical Cure for Intemperance........................... 288
The Phosphates in Nutrition........................... 288

